# Colorectal lymph node harvest in cancer surgery, adequacy and treatment implication: a 5-year retrospective analysis from a tertiary hospital in Ethiopia

**DOI:** 10.3389/fgstr.2025.1503842

**Published:** 2025-03-05

**Authors:** Sirna Emana Jaleta, Abdo Kedir Abafogi, Tamirat Godebo Woyimo, Gashahun Mekonnen Disassa, Sultan Jebel Usman, Abduletif Haji-Ababor Abagojam, Kedir Negesso Tukeni

**Affiliations:** ^1^ Department of Pathology, Jimma University, Jimma, Ethiopia; ^2^ Department of Internal Medicine, Jimma University, Jimma, Ethiopia; ^3^ Department of Surgery, Jimma University, Jimma, Ethiopia

**Keywords:** colorectal cancer, lymphadenectomy, cancer lymph node harvest, pathological tumor staging, Cancer surgeries, lymph node sampling, Jimma Medical Centre, Ethiopia

## Abstract

**Introduction:**

Colorectal cancer is one of the common malignancies, and obtaining sufficient lymph nodes after surgeries is critical for staging and subsequent treatment planning. While guidelines advocate collecting at least 12 lymph nodes, insufficient lymph node sampling can have catastrophic consequences.

**Methods:**

This was a retrospective study that looked at the parameters influencing lymph node retrieval during colorectal cancer surgery in one of tertiary hospital in Ethiopia. In this study, data from 85 patients’ records for stages I-III were analyzed and divided into two groups: adequately harvested and inadequately harvested. The association between potential factors impacting optimal harvests was analyzed.

**Results and discussion:**

The study found that the majority of cancer patients were between the ages of 34 and 53 years, in which the adequate lymph node retrieval was achieved only in 23% of cases. Procedures being performed by GI oncologic surgeons (P = 0.006, AOR;26.4), depth of invasion (AOR:14. P = 0.05), and length of specimen (AOR:5.365 P:0.045) were associated with improved adequacy of harvesting the lymph node. In conclusion, the study discovered that colorectal cancer primarily affects young people. Only a small number of participants had adequate lymph nodes harvested. The operating surgeon’s expertise, tumor characteristics, and specimen lengths were the most important elements influencing lymph node retrieval in colorectal cancer surgery in the setting. Adequate sample length, combined with better availability to more qualified operators, may improve the adequacy of harvest in guiding future treatment decisions.

## Introduction

Colorectal cancer (CRC) is a cancer of the colon or rectum. Cancer begins as a tiny development in the mucosal layer of the colon that is referred as polyps, and are benign (1). Over time, the polyps may develop into malignant (cancerous) tumors known as colorectal cancer (2). It is among the most common types of cancer worldwide, the third most frequent type of cancer in men and the second in women (3). Furthermore, it accounts for 8% of all cancer fatalities, is the fourth leading cause of mortality in Central and Eastern Europe, outpacing tuberculosis, malaria, and HIV/AIDS combined (3, 4). It occurs in hereditary, sporadic, or familial forms (1, 5).

CRC is the third most common malignancy in Ethiopia’s adult population, and patients frequently present in advanced stages of the disease, the symptoms vary depending on the location and stages of the disease (6, 7). Patients may present with symptoms such as bowel habit changes, intestinal obstructions, pain with abdominal mass, unexplained weight loss, blood in the stool, or anemia (7). Furthermore, patients with colorectal cancer on the right side of the colon predominantly exhibit signs of anemia, weight loss, or abdominal pain, while those with cancer on the left side of the colon frequently report an alteration in bowel habit change or rectal bleeding (8).

Surgical resection is the only option for a cure while also providing significant palliation in those with advanced disease. Surgical management should favor adequate resection for cure or palliation rather than bypass or diversion (9). The en-bloc excision of the presenting tumor, including appropriate margins and the lymphatic nodal basin, remains the hallmark of curative surgery. Adequacy of resection is now universally acknowledged to comprise a 10-cm proximal bowel margin and at least a true 2-cm distal margin, together with complete resection of the primary and secondary nodal basin, based on the blood supply of the affected intestinal segment (9, 10).

The involvement of lymph nodes by cancer cells in CRC is a significant step toward systemic tumor dissemination and hence a strong predictor of poor prognosis. Lymph node involvement is a determining characteristic in the AJCC/UICC TNM system, which is now the most important prognostic classification and serves as the foundation for further therapeutic decisions (11–13). Adequate lymphadenectomy and lymph node retrieval from of resected specimen is critical to ensuring staging accuracy, particularly to avoid under diagnoses of lymph node involvement by tumor cells (14, 15). Furthermore, a higher number of sampled lymph nodes has emerged as an independent predictive factor for better survival in several prior investigations, specifically in stage II CRC (14–18).

The current guidelines indicate that at least 12 lymph nodes should be checked to ensure appropriate sampling. Because detecting any positive lymph node is crucial for predicting patient outcomes, a sufficient number of lymph nodes must be investigated as inadequate lymph node sampling has severe consequences. It can result in positive lymph nodes being overlooked and patients being incorrectly categorized as having lymph node-negative disease and hence these patients might be denied the benefits of adjuvant therapy (10). Various studies have shown that the number of retrieved lymph nodes is influenced by a variety of parameters, including surgical radicality and devoted pathological work-up, as well as patient- and tumor-specific characteristics (14, 15). Little information is available in parameters that influence lymph node harvest in CRC resection specimens in Africa are not well documented. Furthermore, the status and characteristics influencing lymph node harvest following colon cancer surgery in Ethiopia are lacking. The purpose of this study is therefore to investigate the factors that influence lymph node harvest during colon cancer surgery at Ethiopia’s Jimma medical Center between September 2018 and August 2023.

## Methods

### Study area, design and period

A hospital-based retrospective study was done at Jimma Medical Centre of Ethiopia in the department of pathology from September 30 to December 20, 2023. The medical center serves about 20 million people in the Jimma zone and southwestern region of Ethiopia. Aside from offering therapeutic services to patients, the Centre offers a variety of undergraduate and postgraduate degrees in basic sciences and clinical medicine. The pathology together with other clinical departments offers cytopathology, surgical pathology, and hematopathology services.

### Inclusion and exclusion criteria

While all patients who underwent resection for colorectal carcinoma during the study period and had a specimen submitted to the pathology department with a histologic diagnosis of adenocarcinoma were included, those with incomplete specimens or pathology reports were excluded.

### Sample size and sampling technique

From September 2018 to August 2023, all sequential colectomy specimens having a histologic diagnosis of colorectal cancer that satisfied the inclusion criteria were identified retrospectively using a non-probability convenient sampling method. This procedure entailed analyzing all biopsy hard copy reports and choosing appropriate cases. Of the identified patients, 95 specimens (81 elective and 14 emergency) with confirmed adenocarcinoma were obtained. Six instances with no lymph node information and four stage IV cases were excluded, leaving 85 cases for analysis.

### Data collection tools and procedures

A comprehensive checklist with sociodemographic, clinical, and microscopic data was developed. The data was extracted by three technical assistants, two first-year pathology residents and one lab technician, following the checklist and under careful supervision and support from the primary investigator.

### Operational definitions

- Colorectal cancer: carcinoma arising from large intestines from the cecum to the rectum
- Right side tumor: tumor located from the cecum to the splenic flexure of the colon
- Left side tumor: tumor located in splenic flexure to distal Rectum
- Tumor histologic type: based on WHO GI tumor classification 5^th^ Edition
- Tumor stage: based on AJCC 8 TNM staging
- Tumor differentiation: based on WHO for 2019 Two-tier grading system
- In adequate LN: LN number <12
- Surgeon level of training: specialty or sub-specialty
- Pathologist level of training: pathology resident or pathologist

### Data quality control

The data collectors received two days of instruction on how to retrieve, categorize, and record data. The principal investigator constantly monitored and guided the data collectors as they extracted and recorded biopsy results from the pathology department archive using prepared checklists. Every day, the principal investigator rechecked each data point for completeness, consistency, and accuracy to ensure data quality. Furthermore, any data that did not satisfy the defined inclusion criteria or was found as incorrect was carefully noted and removed.

### Data processing and analysis

The acquired data was coded and input into Epidata 3.1, then cleaned and exported to SPSS version 26 for analysis. Descriptive statistical analysis was performed, and categorical variables’ results were presented using frequency, percentages, tables, and graphs, while continuous variables were summarized using mean, standard deviation, and histograms. To examine the relationship between independent factors and dependent variables, inferential statistical analysis was performed using bivariate and logistic regression with multivariable analysis. The Chi-square/Fisher Exact test was used to determine the significance of study parameters on a categorical scale comparing two or more groups, with significance set at P value < 0.05.

### Ethical consideration

The Jimma University Institutional Review Board provided ethical approval, and the Department of Pathology granted authorization to conduct the study.

## Results

### Socio-demographic characteristics of study participants

**Figure 1:**
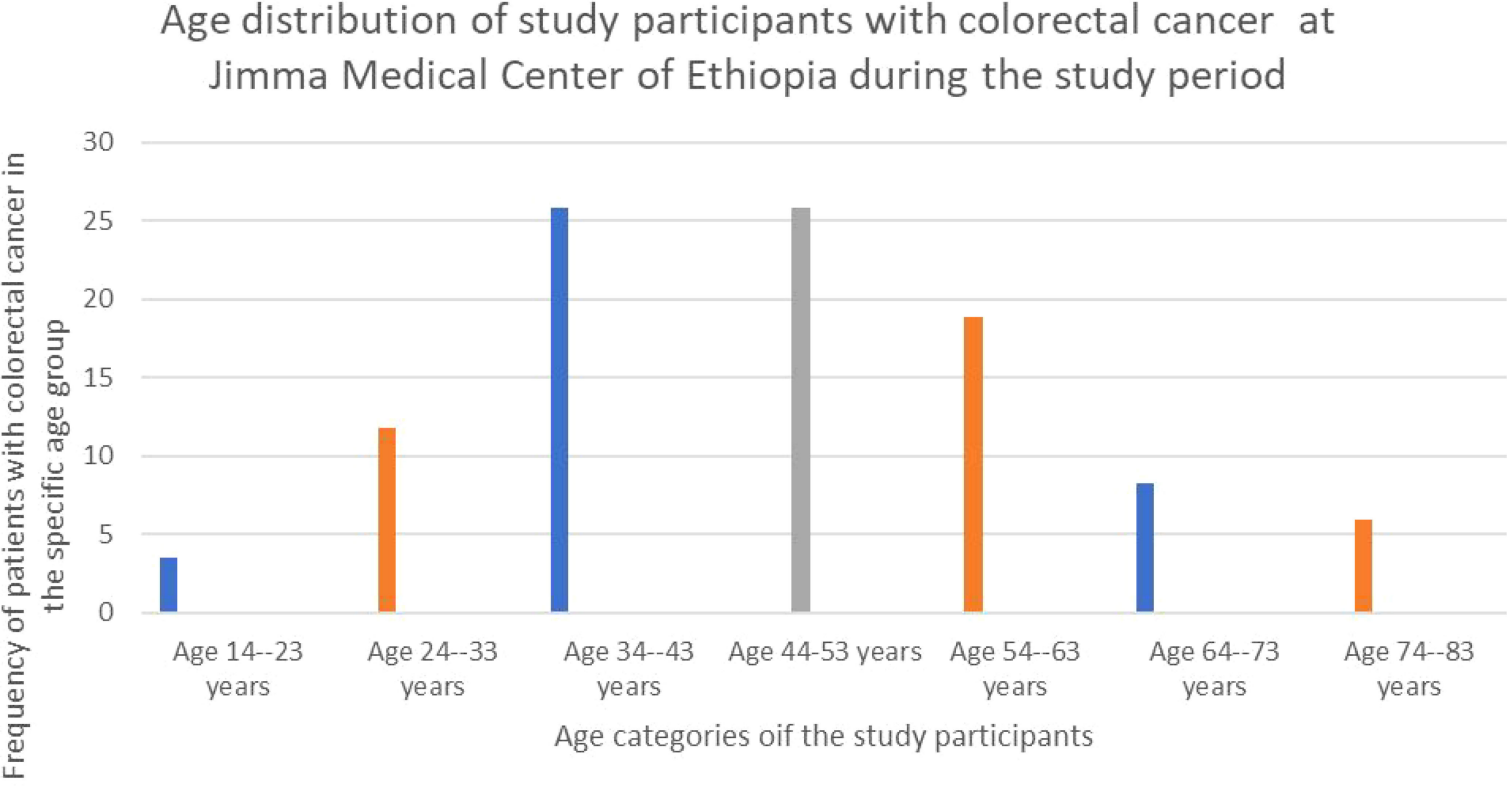
The bar graph showing the age distribution of study participants of patients with colorectal cancer at Jimma Medical Center of Ethiopia, from September 2018 to August 2023.

Out of the eighty-five participants in the study, forty-six (54.1%) was female with a male-to-female ratio of roughly 1:1.12. The participants’ ages ranged from 14 to 80 (average was 47.52 with SD of 15.233) years. Interestingly, the majority 44 (51.76%) were between the ages of 34 and 53 years. The next most common age group was 54-65 years, accounting for 16 (18.82%) participants (**Figure 1**).

### Clinical characteristics of the study participants

Of the eighty-five study participants, sixty-five (76.5%) had reported tumor location information. The sigmoid colon was the most common tumor site among recorded cases, accounting for 26 cases (30% of the total). The cecum was the second most common site, accounting for 16 instances (18.8%). None of the cases had undergone neoadjuvant chemoradiotherapy (**Table 1**).

**Table 1:** The distribution of tumor location and type of resection in study of patients with colorectal cancer at Jimma Medical Center of Ethiopia, from September 2018 to August 2023.

Tumor Location	N (%)
Cecum	16 (18.8%)
Ascending colon	9 (10.60%)
Transverse colon	1 (1.20%)
Descending colon	1 (1.20%)
Sigmoid colon	26 (30.60%)
Rectum	10 (11.80%)
Not documented	20 (23.50%)
Rectosigmoid junction	2 (2.40%)
Type of Resection	
Ileocecal resection	1 (1.20%)
Not documented	24 (28.20%)
Right hemicolectomy	28 (32.90%)
Left colectomy	6 (7.10%)
Total colectomy	8 (9.40%)
Sigmoid colectomy	18 (21.20%)
**Total**	85 (100.00%)

**Figure 2:**
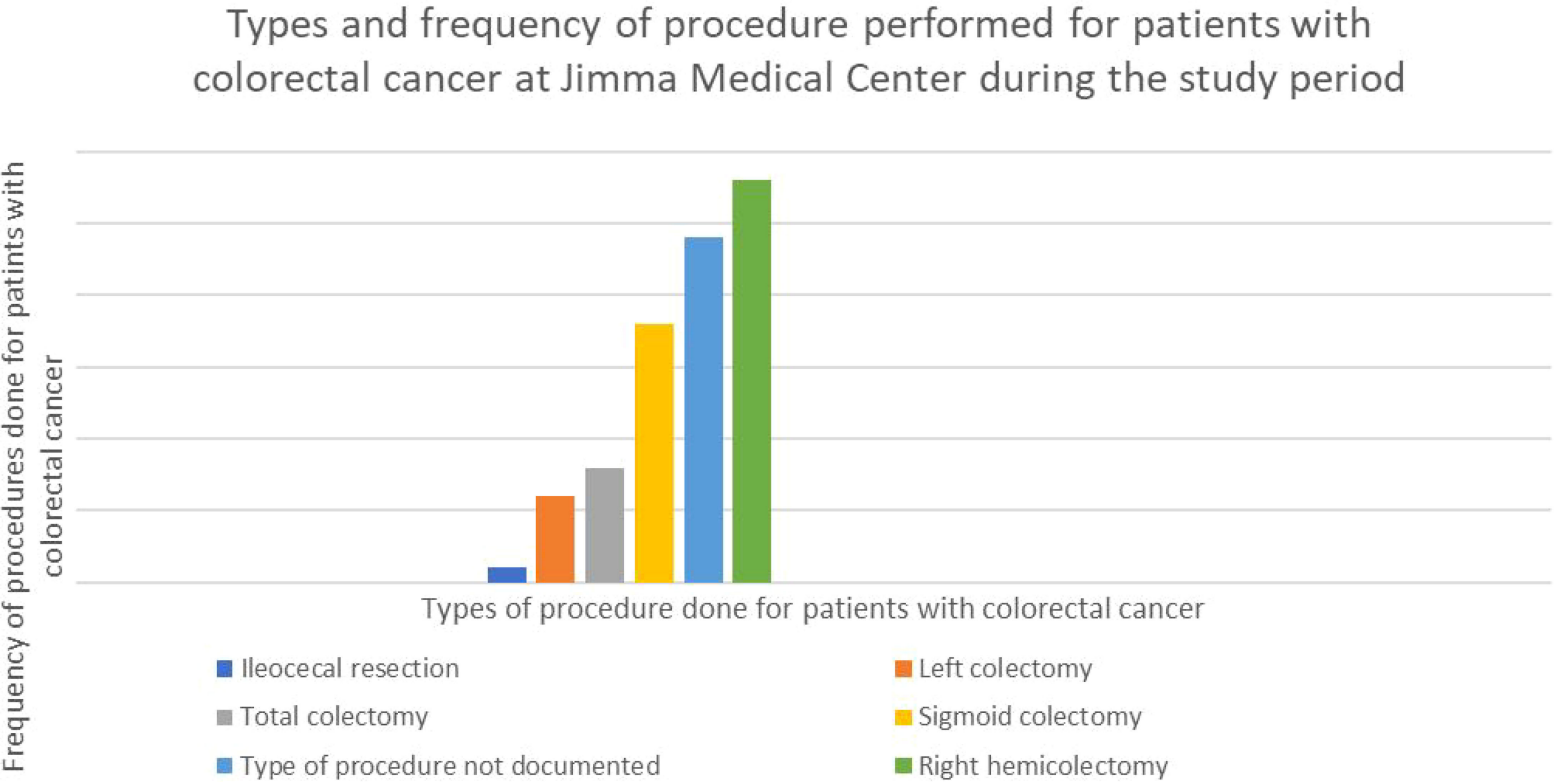
Graph showing the type and percentage of resection done for CRC in the study participants with colorectal cancer at Jimma Medical Center of Ethiopia, from September 2018 to August 2023.

Out of the eighty-five subjects, sixty-one (71.8%) had documented surgical procedures. The most common operation among those with recorded procedures was right hemicolectomy, which was performed on twenty-eight (32.2%) of the patients. The second most common procedure was sigmoid colectomy, which was performed on eighteen patients (21.2 percent) (**Figure 2**).

Among the eighty-five individuals analyzed, the majority (76.5%) had surgery performed by general and colorectal surgeons while all of them had their tissue inspected by pathologists. Furthermore, 83.5 percent of surgeries were elective.

### Tumor size, length of colon resected

**Figure 3:**
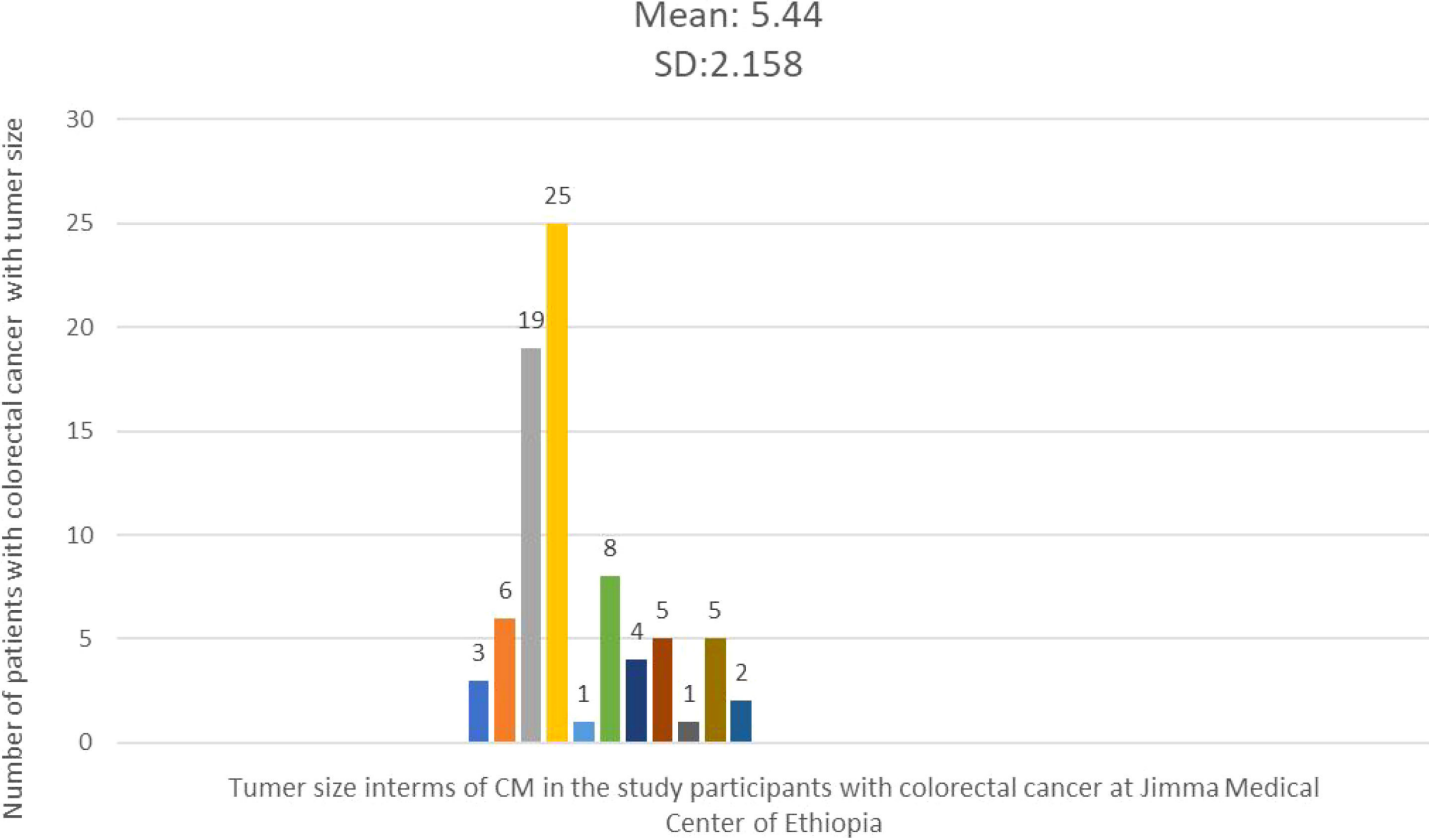
Histogram showing the frequency distribution of tumor size in the study participants with colorectal cancer at Jimma Medical Center of Ethiopia, from September 2018 to August 2023.

The average tumor size in 79 cases with documented tumor sizes was 5.44 cm, with a standard deviation of 2.18 cm. The smallest tumor measured was 2 cm, while the largest was 15 cm. The majority of cases (67 patients) had tumor sizes less than 5.44 cm in diameter (**Figure 3**). The average length of the resected colon was 29.67 cm, with a standard deviation (SD) of 14.725. While the minimum and greatest lengths removed were 10 cm and 80 cm, respectively, the majority of cases (57.1%) comprised colon resections of less than 29.67 cm in length. The right and left colons had average lengths of 39.19 cm with an SD of (15.5) and 24.21 cm with an SD of 13.27, respectively.

### Tumor gross morphology, microscopic report of margin status

Regarding the tumors gross appearance, the majority (63.5%) were classified as polypoid masses, followed by circumferential constrictive thickening (31.8%) pattern. In this study, the majority of proximal (83, 97.6%) and distal (81, 95.3%) resection margins were tumor-free, indicating satisfactory surgical margins in most cases. However, a small number of cases had positive margins, with two cases involving the proximal margin and four involving the distal edge. The analysis of radial margins revealed that more than half (54, 63.7%) were clear of tumor involvement, with only two cases implicated. Furthermore, twenty-nine cases (34.1%) lacked verified radial margin status. In terms of histologic features, conventional adenocarcinoma was the most common type observed in this investigation, accounting for 75 (88.2%) cases. There were eight cases of mucinous carcinoma. The majority of tumors (58, 68.2%) were classed as well-differentiated, whereas just a minor percentage (18, 21.2%) were classified as moderately differentiated (**Table 2**).

**Table 2:** Summary of radial margin, histologic feature and grade, as well as tumor grade among the study participants with colorectal cancer at Jimma Medical Center of Ethiopia, from September 2018 to August 2023.

Feature	Value	Frequency	Percentage
Proximal margin	Free	83	97.60%
	Involved	2	2.40%
distal margin	Free	81	95.30%
	Involved	4	4.70%
radial or another margin	Free	54	63.50%
	Involved	2	2.40%
	Not documented	29	34.10%
histologic type	Adenocarcinoma	75	88.20%
	Mucinous carcinoma	8	9.40%
	Signet ring carcinoma	1	1.20%
	Adenoma like carcinoma	1	1.20%
histologic grade	Well-differentiated	58	68.20%
	Moderately differentiated	18	21.20%
	Poorly differentiated	9	10.60%
pT stage	Depth	Frequency	Percentage
	pT1	1	1.20%
	pT2	33	38.80%
	pT3	39	45.90%
	pT4a	10	11.80%
	pT4b	2	2.40%

### Depth of invasion

**Figure 4:**
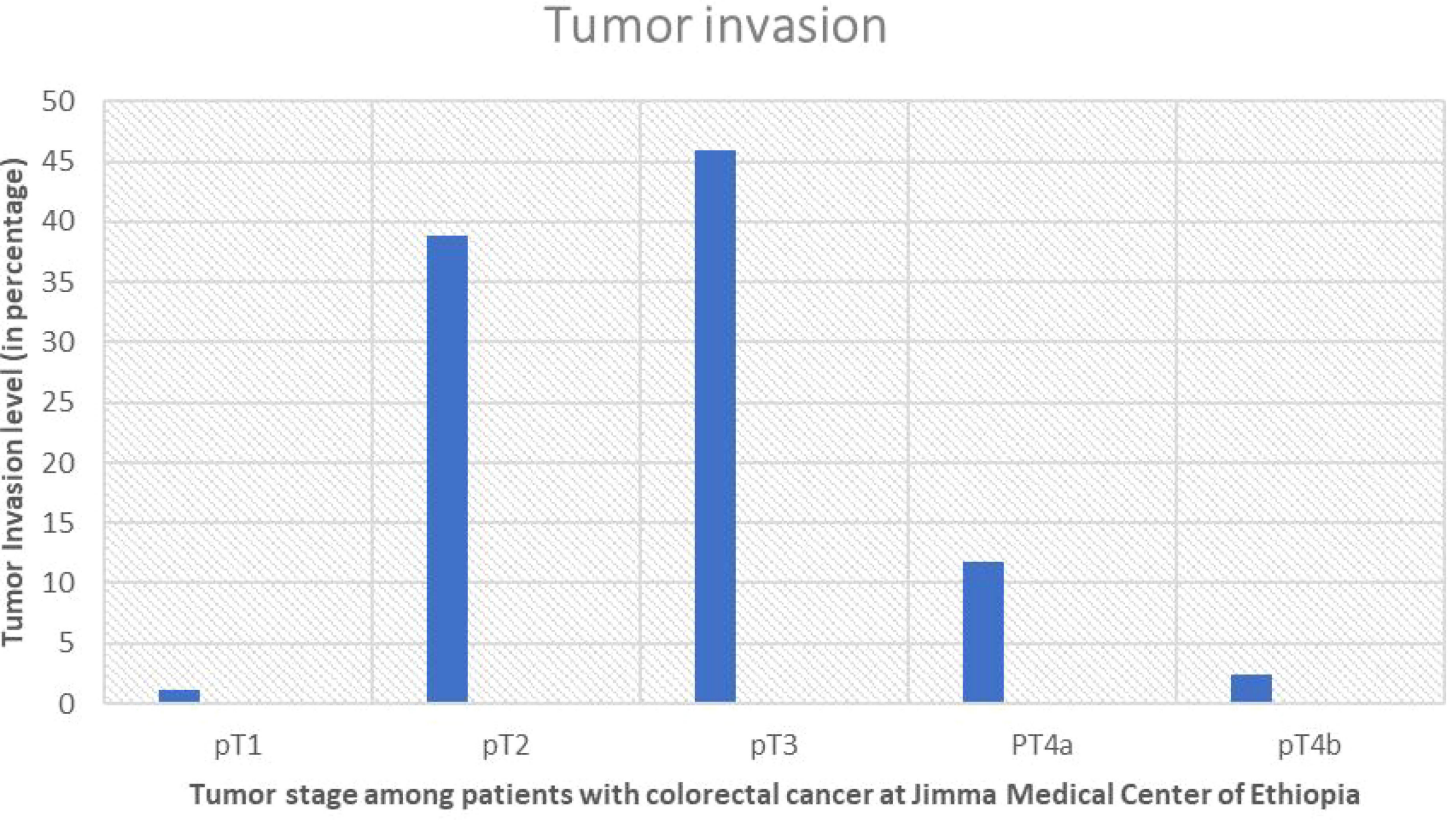
Showing percentage of pT staging and level of invasion of the tumor in study participants with colorectal cancer at Jimma Medical Center of Ethiopia, from September 2018 to August 2023.

**Figure 5:**
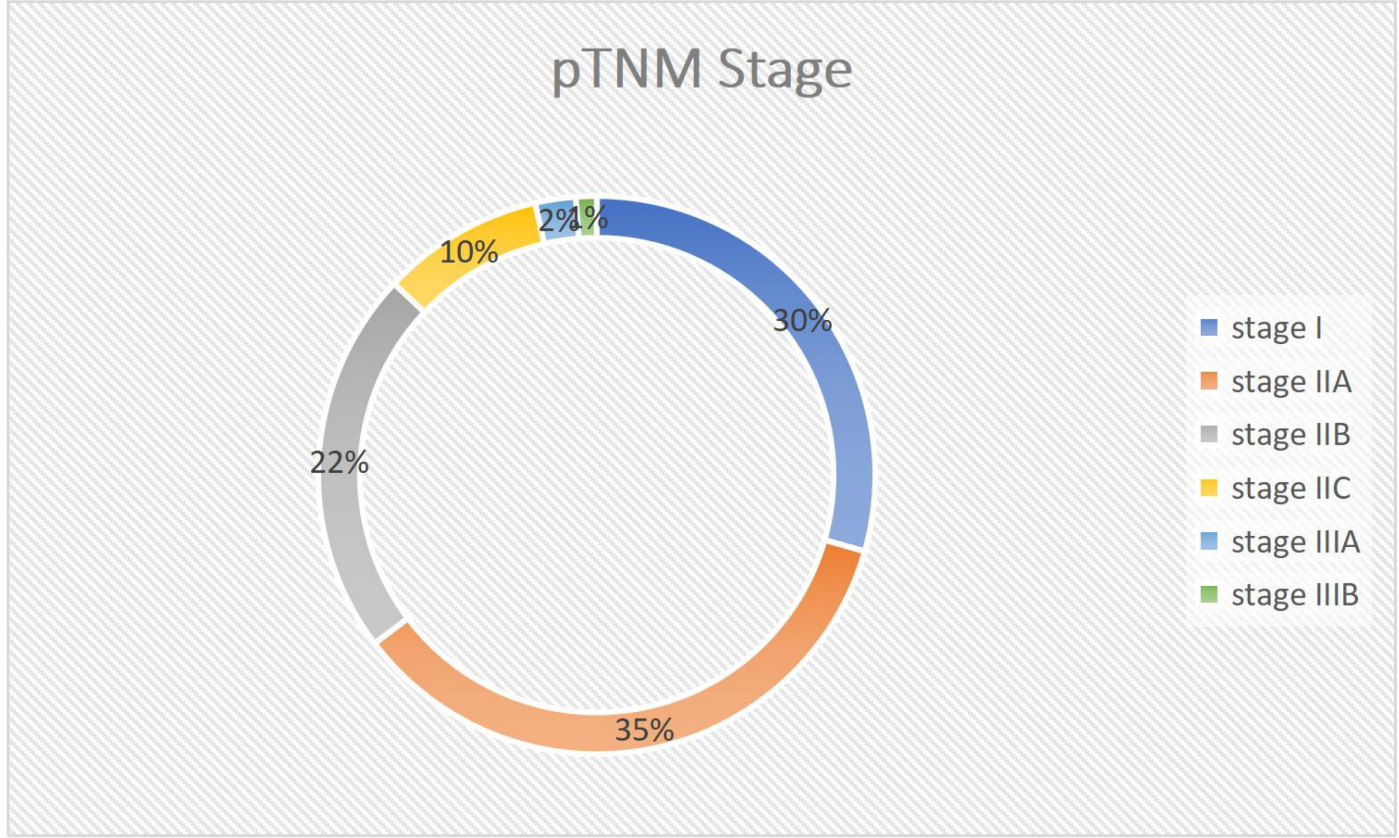
Pie chart showing TNM Stage Distribution of the tumor stage in the study participants with colorectal cancer at Jimma Medical Centre of Ethiopia, from September 2018 to August 2023.

The most prevalent pathologic tumor stage, or depth of invasion to the wall, was pT3 stage, which was found in 39 cases (45.9%), followed by PT2 which also was found in 33 cases (38.8). Furthermore, of the 45 cases with established lymph vascular infiltration (LVI) status, only 20 (44.45%) had LVI (**Figures 4**, **5**).

### Pathology report of number lymph nodes

This study found an average of 7.01 lymph nodes removed following surgery, with a standard deviation (SD) of 4.79. The smallest number of lymph nodes retrieved was zero, while the greatest was 18. The majority of cases (61.2%) had less than 7.01 lymph nodes detected. The average number of extracted lymph nodes grew annually, from 4.5 ± 3.5 SD in 2011EC to 8.94 ± 5.9 SD in 2014. The mean of lymph nodes extracted varied significantly with different predictors, which were highest when the surgery was done by a GI surgery fellowship trained surgeon and grossing done by a pathologist (9.33 ± 4.3 SD) and lowest when the specimen was shorter (below 30CM) (5.41 ± 4 SD).

The average size of the largest lymph node found was 1.5cm ±0.6 SD. However, only 20 cases (23.5%) were classified as sufficiently harvested lymph nodes (identified LN>11). The most common pathologic stage is IIA (35.3%), followed by stage I (29.4%). Adequate lymph node harvest was only achieved in 8 of 30 stage IIA cases and 1 of 25 stage I instances.

### Factors influencing lymph node harvest

The age, depth of tumor invasion, tumor laterality, surgeon’s level of training, histologic grade, tumor size, lymph node positivity, and specimen length were factors influencing the lymph node harvest all with (P-value < 0.05). There were no significant associations found for tumor size, sex, or size of the biggest lymph node (**Table 3**).

**Table 3:** Factors affecting analysis of lymph node adequacy in study in the study participants with colorectal cancer at Jimma Medical Center of Ethiopia, from September 2018 to August 2023.

Variable	OR	At 95% CI	P-value
Tumor-negative lymph node	0.622	0.375 - 1.034	0.622
Tumor positive LN	1.986	1.137 - 3.47	0.024
Procedure: General Surgeon	0.709	0.489 - 1.029	0.709
Procedure: GI surgeon	2.6	1.188 - 5.689	0.015
Specimen ≤30cm	0.487	0.243 - 0.978	0.487
Specimen >30cm	1.82	1.195 - 2.772	0.013
Depth invasion ≤pT2	0.433	0.174 - 1.082	0.433
Depth invasion: >p2	1.486	1.085 - 2.034	0.037
Age: ≤50	1.486	1.085 - 2.034	0.03
Age: >50	0.433	0.174 - 1.082	0.433

CI, confidence interval; OR, odds ratio; LN, lymph node; GI, gastrointestinal; pT, pathological tumor staging.

Binary logistic regression was used to assess lymph node adequacy and independent factors that had significant relationships in the univariate study. The data revealed that GI oncologic surgeons (26 times more likely), deeper tumor infiltration (pT2 and above, 14 times more likely), and longer specimens (>30 cm, 5 times more likely) significantly increased the likelihood of attaining appropriate lymph node harvest (≥12 nodes) in colon cancer patients. However, age over 50 was found to be a negative predictor of adequate harvest (87% reduction in probabilities). Notably, there was no significant link between tumor laterality, lymph node positivity, and sufficient harvest (**Table 4**).

**Table 4:** Shows a multivariable logistic regression analysis of adequately harvested lymph nodes and associated variables in the study participants with colorectal cancer at Jimma Medical Center of Ethiopia, from September 2018 to August 2023.

Variables	Adequate LN harvested (%)	Inadequate LN harvested (%)	Exp (B)	95% C.I. for Exp(B)	P value
Depth invasion > pT2	16(31)	35(69)	2.704	14.942,101.399)	0.006
Length of specimen > 30 cm	14(35.8)	25(64,2)	5.365	(1.041, 27.644)	0.045
Age > 50	4(11.7%	30(88.3)	0.126	(0.025, 0.644)	0.013
High-grade tumor	–	–	3.42	(0.378,30.945)	0.274
LN positivity	–	–	0.895	(0.158, 5.076)	0.901
Right side tumor	–	–	0.988	(0.151, 6.474)	0.99
GI surgeon	10(50)	10(50)	26.402	(2.613,266.737)	0.006
Constant	-1.722		0.179	(0.027,1.329)	0.296

## Discussion

Inadequate lymph node sampling has severe consequences. It can result in positive lymph nodes being overlooked and patients being incorrectly categorized as having lymph node-negative disease. Such patients may not have the opportunity to benefit from adjuvant therapy. Furthermore, inadequate lymph node sampling may fail to remove relevant lymph nodes, increasing the likelihood of local recurrence; it may also be a sign of poor surgical or pathologic care, both of which are linked to poorer long-term results for colon cancer patients.

This study aimed to identify parameters influencing lymph node retrieval of at least 12 LNs from patients with CRC as specified by AJCC recommendations. In this study, 85 patients who met the criteria were enrolled. The study population had a mean age of 47.52± 13.08 years, with the range from fourteen to eighty. The majority (51.76%, or 44 individuals) were between the ages of 34 and 53 years. According to study in Addis Abeba, the average age was 47.52 ± 16 (SD), with 36% of the population under 40 (19).

Our study found a high percentage of colorectal cancer in people under the age of 40 years (38.8%), which is much higher than the European (3%) (20) and US (20%) rates. However, this is consistent with the study (19),which identified 36% of patients who were under-40 years. This study was also similar with that of Nancy J’s research in India (21), which found 35.5% of young adults with CRC. Furthermore, the mean (SD) number of LNs extracted was 7.01 ± 4.8, with a range of 0 to 18 LNs, that was comparable to the Wright et al. research in Canada 2003, which revealed a mean of 7.0 LNs, and slightly lower than another study (22) who exhibited a mean of 8.3 LNs. However, it was much lower than other studies (10, 19, 23),which reported a mean of 9, 10.1, and 11.7 harvested LNs, respectively. This was also lower than two studies in Nepal (24), which found a mean of 9.8 LNs, and 14.5 LNs, respectively. This could be from the fact that both of them did not include rectal cancer, which has been associated with a lower rate of LN harvest compared to colonic cancer in different studies.

This study found that only 23% of patients achieved acceptable LN harvest (≥12), which is slightly lower than the percentages reported by the other studies. Furthermore, GI surgery fellowship training was found to be strongly related with adequate lymph node harvest, with a 26-fold increase in the likelihood of being adequately harvested. This is consistent with one study (25), which found an adequacy rate of (77 vs. 63) for fellowship-trained surgeons and general surgeons. However, the bigger discrepancy in this study could be attributed to the smaller sample size. Furthermore, specimen length was substantially related to proper lymph node harvest, which was consistent with another studies (26, 27). This makes sense, as a longer colon segment has more lymph nodes holding mesocolon, leading in a higher yield.

Our study, like others (10, 28–32), indicated that patients under 50 achieved considerably higher sufficient lymph node harvest (≥12 LNs). This could be owing to a stronger immune response in younger people, making the nodes more visible to surgeons and pathologists. Additionally, age-related involution of lymphoid tissue may contribute to reduced harvest rates. While these are plausible theories, more research is required to fully comprehend the intricate interaction of factors impacting LN recovery.

This study found a substantial correlation between the depth of tumor invasion and the number of lymph nodes retrieved. This is consistent with previous study, which suggested that deeper penetration of the gut wall in T3 and T4 tumors causes a larger antigenic immunological and inflammatory response inside the nearby lymph nodes, making them more visible during pathological examination (10, 31–33). In a population-based study, one study found that the T stage was independently linked with the number of investigated LNs (33). Similarly, research conducted in India and other one (24, 34–36), in the Netherlands indicate that advanced TNM stage can result in larger LN size, making them simpler to recognize and thus contributing to higher LN harvest.

Our results did not show a statistically significant relationship between lymph node yield and tumor size. This is also consistent with a single-institution study in Nepal which enrolled 87 study participants and found no relationship, in contrast to data from multiple other studies indicating a higher yield of lymph nodes in patients with bigger tumors (10, 22, 31, 37). This could be due to the fact that the majority of research that found a favorable relationship between tumor size and lymph node yield focused on rectal cancer. Furthermore, the cancer location was more diversified in our study, which could lead to a weaker correlation. The small sample size may have hampered our ability to detect a statistically significant link as well. As a result, more study with bigger and more diverse patient populations is required to completely understand the complex link between tumor size and lymph node yield in colorectal cancer.

Unlike prior studies (10, 30, 31, 38, 39), this study was unable to reveal a link between tumor site and lymph node harvest. This gap could be explained in part by the presence of a high number of unrecorded cases (15%) at the sites. More study with larger data sets and various patient populations is required to determine the true association between right colon tumor site and lymph node yield. Current study on the association between lymph node (LN) positive and lymph node harvest (LNH) yield produces inconsistent results. This study revealed no significant link between the two, which is consistent with other studies done elsewhere (39).

However, some studies (22, 23), reported opposing findings, indicating a positive relationship between nodal positivity and increased LNH yield. This could be because specimens with numerous LN metastases were not as thoroughly tested for further LNs. Another possible reason is that our small sample size is insufficient to reveal a genuine association. As a result, further research with a large sample size is needed to investigate the association between lymph node positive and appropriate lymph node yield. Similarly, this study revealed no statistically significant correlation between histologic subtype/grade and lymph node yield, unlike some earlier investigations (22–24). This could be owing to the small sample size in our study as well, and more research with a larger population size is required to demonstrate the association between histologic/grade and lymph node adequacy. Previous investigations (10, 15, 23, 24, 26), discovered that pre-operative chemotherapy or radiotherapy has a significant effect on lymph node yield, which could not be measured because all of the patients had no history of prior treatment in this study.

### Limitation of study

Our findings provide light on factors influencing colon cancer lymph node yield, but limitations necessitate additional research. Its small size and single-institution setup limit generalizability, while inadequate data hampered the assessment of particular characteristics such as tumor location. Nonetheless, this study establishes the foundation for future research with larger, more comprehensive datasets to definitively unravel the complex interaction of variables influencing lymph node yield in colorectal cancer.

### Conclusion

This study provides an overview of the incidence, prevalence, and age/gender distribution of colorectal cancer at a tertiary hospital center in Ethiopia. Through a thorough retrospective data analysis, we evaluated the appropriateness of lymph node sampling, which has a significant clinical and prognostic impact on patient outcomes. Based on the results, we emphasize the importance of obtaining adequate sample sizes that accurately represent lymph node status. An additional recommendation is to involve qualified, when demanded additional trained operators. This is expected to enhance the quality of lymph node sampling. This, in turn, could substantially improve future treatment prospects and clinical decision-making.

## Data Availability

The original contributions presented in the study are included in the article/supplementary material. Further inquiries can be directed to the corresponding author.
